# Medical Notes on China

**Published:** 1846-07

**Authors:** 


### Art. XV.

*Medical Notes on China.* By John Wilson, m.d., f.r.s., f.s.s., Inspector of Naval Hospitals and Fleets.—*London,* 1846. 8vo, pp. 267.

We have on several occasions brought under the notice of our readers the valuable Reports on the Health of the Navy, compiled by Dr. Wilson, from the official returns of the medical officers of that branch of the service. The volume before us from the pen of the same author is of a different character, being rather a medical history than a statistical account of the naval portion of the Chinese expedition. The French possess many excellent works of this description, but their utility appears hitherto to have been much overlooked in our service. It seems highly desirable that when an army takes the field, the senior medical officer with it should be directed to collect the necessary information, and at the end of the campaign to draw up a general account of the diseases which have prevailed among the troops, their apparent causes, the measures adopted to preserve the health and efficiency of the men, and the success which has attended these measures. Similar instructions might be given to the senior medical officer of the fleet in cases where a naval force is employed. To enter fully upon the advantages which might be expected from a regulation of this nature would occupy more space than we can at present afford, we would merely remark, that had Sir John Pringle's work, which might well be taken as a model for such reports, been studied by the authorities in 1809, very many men might have been saved who fell inglorious victims to the Walcheren fever.

In March, 1842, the Minden, 72, Captain Quin, which had been fitted up as an hospital ship, calculated to accommodate 200 patients, besides the crew, and to which Dr. Wilson had been appointed principal medical officer, with an adequate staff, was sent out to Chusan, where she arrived on the 15th of August. No pains or expense had been spared to render her in every respect suitable for the purpose for which she was intended. "Nothing," says our author, "was omitted which, in the opinion of the authorities who ordered and designed the hospital establishment of the Minden, could conduce to the comfort, convenience, and well-being of the sick and wounded who might be received into it; and it may be unhesitatingly asserted that no such moveable hospital, in respect of magnitude, means of efficiency, and completeness, ever left an English port."

From the period of his arrival at Chusan, Dr. Wilson noted "from time to time, whatever appeared to him most worthy of record in China: first in respect of disease, its nature, treatment, progress, and results; and second, regarding its causes, apparent and probable, whether permanent and necessary, or artificial and capable of being removed." These notes, extending over a period of above two years, from August 1842, to the end of 1844, constitute the volume now before us, and are printed as originally put down without any alterations. We cannot but regret that our author adopted this form of publication in preference to a more finished and systematic work founded on his notes, and that he has not given us more information of a statistical nature, which, from his previous pursuits and acquaintance with the subject, he was so well qualified to collect. The meteorological observations also, which appear to have been made with great care, would have been much more useful if tabulated, than dispersed as they at present are throughout the book. It is but just, however, to mention that during a considerable portion of our author's service in the China seas his health was much affected, and it is most creditable to him that, notwithstanding the bodily suffering and languor under which he laboured, he continued to collect so much interesting and valuable information as is contained in the work before us.

We have already stated that the "Notes" extend over a period of upwards of two years; during the first nine months the Minden was in the harbour of Chusan, and for the rest of the time in that of Hong-Kong.

I. At the date of the arrival of the Minden the principal part of the force was employed in the Yang-tse-Kiang, whence the most severe cases of disease were sent to Chusan, as opportunity offered, for treatment on board the hospital ship.

On the 1st of July, 1842, the numerical strength of the naval force was about 4200 men; during the quarter ending 30th September, the number entered on the sick lists amounted to 5201, or 124 per cent. of the strength; and the deaths to 88, or $2\frac{1}{10}$ per cent., while the proportion of deaths to the number treated was one in 59.*

The diseases by which this enormous amount of sickness (the admissions during one quarter being a fourth more in number than the whole force employed) was occasioned, were principally fevers and diseases of the bowels; of the former, 1951 cases occurred, whereof 1644 were intermittents, and of the latter, 1606 were entered in the sick returns, of

* Among the ships composing the "Home Force," the annual ratio of admissions into hospital amounts to 120 per cent., and of deaths to 1·07 per cent. of the strength. Consequently, in the Chinese fleet, the sickness in three months was rather more, and the mortality double what would have occurred in twelve months among the men employed at home. It must, however, be remembered, that the three months in question constitute the sickly season there. We have unfortunately no statement of the number of admissions and deaths during the rest of the year, but there can be no doubt that, although less than that noted above, it would still be found very considerable.

which 1086 were diarrhœa, and 226 dysentery. The last number, however, is by no means a correct index of the amount of dysentery which came under treatment, because cases admitted as diarrhœa often ran into that form, and it also frequently attacked patients labouring under intermittent fever. This indeed formed one of the principal peculiarities in the fevers of these shores, and materially increased the difficulty of treating them.

"With very few exceptions the periodic fevers and fluxes have been complicated with each other, often coexisting, but more frequently alternating; and this complication has made a principal difficulty in adjusting the means of treatment; for those fitted to be useful, and promising utility in the fever, having generally proved injurious in the flux; it happens constantly that, on the subsidence of the former, the latter sets in to yield in its turn, and again to recur, till the subject is worn out, the exhaustion being generally associated with an immense amount of organic injury." (p. 53.)

"Here the flux ought to be considered a constituent part of the periodic fever, rather than an independent malady in most instances, but is often so prominent a part, and presses so closely on the attention both of patient and practitioner as greatly to distract the observer, and disguise the original essential affection. The obscurity thus occasioned is apt to be increased by the conflict of indications in treating the two phases on the supposition that it is but one and the same in reality; for as has been stated above, and is pretty clear from the nature of things, the means which are fitted to act remedially in the intermittent fever, often cannot be borne, or act injuriously in the intestinal flux." (p. 58.)

Another very troublesome disease which appeared intimately allied to fever, and prevailed to a very considerable extent, was sloughing ulcer. It frequently happened in patients admitted on this account that as soon as the ulcer began to heal favorably, periodic fever appeared, and alternated with the ulcer, or *vice versa,* leading to the belief that, in many cases at least, they were caused by the same morbific influence. This occasional identity of origin is to a certain extent corroborated by observations made in the West Indies, where, during epidemics of remittent fever, soldiers affected with ulcers generally escaped an attack.

The fever at Chusan was usually of an adynamic character, and bore reductive treatment badly. Sometimes a sudden and unaccountable sinking of the powers of life took place.

There can be little doubt that the fever and dysentery were the product of marsh miasm. In the Island of Chusan no natural marshes exist, but every spot of ground capable of being irrigated has, with great perseverance and ingenuity, been converted into a rice field, or in other words, an artificial marsh. "If heat, moisture, and vegetable matter excite periodic fevers, as they evidently often, but not always, do, few places can be supposed more prolific of their cause than the city and plain of Tinghae," the capital of Chusan. After taking a review of the topography of the island and nature of the seasons, Dr. Wilson says—

"The meteoric influences and the aspect of the country appear highly favorable to health. What is detrimental is believed to be chiefly the wilful work of man's hands, or of his neglect, and perverse ignorance. If he acted his part intelligently and well, there is little doubt that this would be one of the most salubrious, as it is naturally one of the most favoured portions of the earth's surface. It would not be becoming to assert that ague, flux, and ulcer could be entirely banished by any human means; but it may be alleged confidently that they might be much reduced both in frequency and force, if the inhabitants would abandon some of their agricultural and economic usages, supplying their place by other methods simple in themselves, and at once more easily practised and more beneficially operative." (p. 113.)

The same source of endemic disease, the malarious exhalations from the irrigated rice fields, exists to a still greater extent on the shores of the Yang-tse-Kiang, where, during this period, the sailors were chiefly employed, as the flat nature of the country is more favorable for this species of agriculture.

"Here there appears to have been abundant materials for the production of periodic fever. . . . . . The wonder is that with such high degrees of heat acting on such a soil, they were so generally some well defined type of intermittent; cases of remittent fever with much precipitancy and fatal force were not nearly so numerous as might have been expected; indeed they were very rare." (p. 51.)

II. On the 7th of June, 1843, the Minden arrived at Hong-Kong. The diseases experienced here among the sailors were of the same general character as at Chusan, being chiefly endemic fever and dysentery. There were, however, several points of difference worthy of remark, and there was almost complete exemption from sloughing ulcer, which, as already stated, had been found so troublesome.

"It was noted as a subject of some surprise in the north, considering the atmospheric heat and excess of artificial miasmated soil, that remittent fever should have been so rare, the fever there so constantly assuming the intermittent form, and generally, however, complicated with other morbid actions, exhibiting regular and well defined types. Here, on the contrary, there have been during the last three months (June, July, and August, 1843) a large proportion of remittent fever frequently, it is true, as elsewhere, when not terminating fatally, issuing in ague; but the agues have not been so formal in type as at Chusan; and there [this?] is another well-defined point of difference between the morbid manifestations of the two places. Then there is a striking feature of likeness between them, that, namely, of their being in both places associated with flux; for here, as there, the two forms of disease—fever and flux—are often so intimately linked together that it is difficult to tell which is the primary, or ought to be considered the principal affection. Perhaps fever in a majority of instances appeared first, but the exceptions have been so numerous, fever being so often preceded by flux as to give it little claim to be considered a prevalent or necessary priority. Whichever appeared first it constantly happened that as one series of morbid actions declined the other rose. They were sometimes concomitant, but more generally appeared in rotation, whatever the order of precedence and of succession might be." (p. 130.)

The sensorium was generally so much affected in the fever at Hong-Kong as to procure it the name "head fever" among the men.

On examination, post mortem, the following were found to be the most constant and striking appearances:

"In the worst cases [in which reaction did not take place, but the patient died in the invasive congestive stage] nothing was detected excepting a loaded state of the large venous trunks, and a certain degree of venous congestion of dependent parts in the thorax and abdomen. The brain and its investing membranes were more exsanguined, and consequently paler than in normal condition, there being no serous effusion either under the meninges or into the ventricles. Neither here nor in other parts was there anything which, with the utmost latitude of language, would be considered inflammatory, or having the least approach to it. The vascular condition of the tissues was, indeed, the very reverse of that which constitutes inflammation."

"When the disease was more protracted, became distinctly paroxysmal, and did not terminate in death for some days—from five to fifteen—the organic appearances had a similar pervading character, but were to a certain extent modified with additions. What could be ascertained of abnormal in structure, was in these, as in the former, with little exception, congestive, not inflammatory, especially manifested in the lungs.......When fever had followed flux, which was not uncommon, ulceration was occasionally found in the colon, the result evidently of the preceding affection. The integrity of organs, including the liver and spleen, when the violence of paroxysms in many cases is considered, is surprising." (p. 135.)

From a consideration of the morbid phenomena and the pathological appearances, Dr. Wilson concludes that the fever was an idiopathic affection, and not symptomatic of local disease; that it was "primarily and emphatically a disease of the whole system, though it might not affect every part of it equally at the same instant;" and that it was the product of pestilential miasm, acting in many respects like a concentrated poison.

On comparing the remittent fever observed in China with the proper endemic of the West Indies, our author observes that there is much resemblance

"In the apparent want of consent between the real force of the affection and its external appreciable manifestation. Here, as there, when there is no complaint made or admitted, and while there is no violence in the febrile signs, it not unfrequently happens that the subject is suddenly seized with hurried respiration, fluttering pulse, partial unconsciousness, or slight coma—a condition which speedily terminates in death. But while there is this point of agreement, there are many points of difference between the two forms of fever. It would be tedious to go over the whole, which, taken together, form a distinct line of diagnosis, but one may be noted in passing. In West Indian fever, death generally ensues, or convalescence is fairly established in a few days; for the most part, within a week and when established it proceeds, steadily in most cases, speedily to completion. Relapse is remarkably rare, and the subject in a short time is as well, strong, and little liable to disease as he was before the attack. In the Hong-Kong fever, on the contrary, when it is subdued, its duration being very uncertain, intermittent fever or flux, as already stated, constantly sets in. There is great proneness to relapse; and after the disease has been apparently subdued, and the tendency to recurrence overcome, the subject continues long listless and emaciated, has a sallow countenance, with pale lips, and hovers on the verge of jaundice, dropsy, or fatal flux." (p. 133.)

The prevalence of fever at Hong-Kong is to be attributed to the same cause as at Chusan, and in the Yang-tse-Kiang, marsh miasm. Notwithstanding the rocky character of the island, rice cultivation was pushed to as great an extent as possible; not only were the level grounds applied to this purpose, but the sides of the hills and ravines were cut into terraces, and by dint of manure and irrigation, the latter being accomplished by means of dams and sluices, were converted into artificial marshes. Since the occupation of Hong-Kong by the British these rice fields have been much neglected, and have in many places fallen out of cultivation—a state in which they are even more injurious and more active sources of endemic disease. To this circumstance Dr. Wilson chiefly attributes the greater intensity and the type of the fever here:

"May it not be inferred," he asks, "that the exhalations from cultivated rice-ground saturated or covered with water at times, but under a regular process of management, and yielding the healthy products of vegetable growth, give rise to intermittent, while the stronger poison exhaled from marsh land, through processes of rapid and multiplied destruction of vegetable matter, under equal degrees of heat, and other apparent circumstances, occasion the more concentrated and fatal remittent fever?"

It is much to be regretted that no steps were taken for the removal of such evident causes of disease. The barracks for the troops have been erected in the immediate vicinity of some of these swamps, and as might have been anticipated, the soldiers have suffered from endemic disease in a much higher ratio than the naval force. There may have been cogent reasons, political or military, for the selection of such a site, but, in the name of common sense and humanity, why was nothing done to render the spot more healthy; or if common sense and humanity are to go for nothing, why was not a system of draining adopted as a measure of economy?

"A few workmen," says our author, "with spades in their hands, under the simplest direction, would do all that is required, at any rate for present benefit, and the benefit would probably be most important; nay, in many places, all that is necessary is to remove the artificial obstructions that have been formed to the natural flow of the surface-water—a practice common in China, and consonant with Chinese notions of salubrity, as well as of productiveness, but which should surely not be permitted now in Hong-Kong. There is a considerable portion of the surface close to West Point, where barracks were built, and last year abandoned, on account of the ravages of periodic fever, which might be rendered inoffensive to the sense, and probably innoxious to health, in a few days, at the expense of a few dollars; and there are many such places in the neighbourhood." (p. 187.)

If we consider that to replace each soldier, who has fallen a sacrifice to fever arising from this neglect of the most ordinary precaution, costs the government not less than £100, at the very lowest estimate, we may safely conclude that this has been, to say the least of it, a most expensive economy of dollars.

The amount and intensity of disease was much less in the hot season of 1844 than the preceding year, without any obvious reason for the difference. This is a very common case in countries where the prevalent diseases are principally of endemic origin, a comparatively healthy season succeeding a sickly one without any apparent cause by which to account for the circumstance. Adverting to this peculiarity our author judiciously remarks:

"On such a difference as this is often raised many a lofty but indifferently founded pretension to improved methods of treating disease; and the vaunted superiority has its origin less, it is believed, in want of candour, than inattention to, and ignorance of, the dissimilar force with which the same disease acts at different times. A practitioner arriving here in the summer of 1844, would find a much larger proportion of the sick under his care recover than did the practitioner of 1843; he would probably, with a laudable desire to accomplish more than the men earlier in the field had done, modify the means of treatment used by them or apply something different; and to this modification or alteration of means, not to the comparatively slight morbid impression, he would be apt to ascribe his better fortune, counting it the effect of professional merit, not of changed circumstances. Having satisfied himself of this, he, and some others who are prone to believe much and to hope much, readily arrive at the conclusion that his predecessors were inefficient practitioners; that they did not understand, or did not properly perform, what was required of them by the obligations of their office, and that they are therefore chargeable with the heavy crime of having allowed men to die who might have been saved." (p. 189.)

Surely, such considerations, backed as they are by facts, should lead us to judge more leniently of the want of success which may occasionally attend the practice of any of our professional brethren, and to look with distrust upon the more fortunate results which may, perchance, follow our own treatment.

We have hitherto noticed the pathological appearances in the fever cases only; the following is a brief description of those usually found in the men who died of dysentery at Chusan:

"Ulceration has been all but universal, and obviously in most instances of long standing, the ulcers being scattered more or less thickly over the whole of the colon and rectum, of various forms and superficial dimensions, generally circular, and from the diameter of a split-pea to that of a shilling or more, the surface being purulent, hemorrhagic, ragged, or clean and smooth, like the effect of excision; of various depths, sometimes destroying the mucous tissue only, sometimes the muscular also, being generally arrested by the peritoneal, but occasionally perforating it. In most cases the rectum has been much thickened and indurated, in many assuming a semi-cartilaginous character; in other cases it and the colon have been attenuated in some places while they were thickened in others; and in both at spots free from absolute ulceration, the mucous lining has been loose and lacerable or abraded, frequently there has been serous infiltration of the peritoneal covering, the mucous lining having a leaden hue, through which light-coloured spots were sometimes interspersed, giving it there the appearance of dark marble; occasionally it had a purple colour, with a rough granular surface, resembling the exterior of a mulberry; and it has not unfrequently happened that the whole tube has been so much softened, broken down, and disorganized as to tear on the slightest touch, or fall to pieces on being lifted. With scarce an exception, the organic lesions have been limited by the ilio-cæcal valve, the small intestines and stomach presenting a perfectly healthy appearance, although venous congestion, and small spots, denoting increased vascular action, have been found occasionally. When it is said that the small intestines and stomach presented a perfectly healthy appearance, it should be added that portions of the former, generally the distal extremity of the ileum, were often, in cases of long duration, much attenuated, and pale, and diaphanous like a prepared colourless membrane." (p. 56.)

Dysentery presented the same general features at Hong-Kong as at Chusan, but was more frequently attended with some affection of the stomach and small intestines.

"Vascular patches, occasionally diffused inflammation tending rapidly to disorganization, often appear in the stomach; similar appearances are found in the small intestines, where ulcerated spots have besides been detected. The numerous autopsies which have taken place show that the primary and permanent source of the intestinal symptoms has been in the large, lower portion of the tube; but it is not, as was almost universally the case at Chusan, confined in a great degree to the ileo-cæcal valve, the cæcal portion of the colon and rectum. On the contrary, the transverse and descending portion of the colon as well as the rectum are deeply involved; being livid, thickened, indurated at one point, softened at another, abraded, and ulcerated, ulceration occurring in small section-like points, or deep excavations, often penetrating to, occasionally through, the peritoneal membrane, which is generally tumid from serous infiltration. The rectum is hypertrophied and ulcerated in an especial degree, and in some instances there is a great amount of vascular turgidity and irregular thickening of a mulberry appearance on the mucous surface, from which, and through the ulcerous openings, the unmixed blood and brown-coloured homogeneous dejections which occurred soon before death, occasionally, were supplied." (p. 139.)

One of the most remarkable circumstances connected with the dysentery of China was the complete absence of diseased appearances in the liver. Considering the frequency with which hepatic disease occurs in India in conjunction with dysentery, this fact is very striking. After three months' experience at Chusan, Dr. Wilson observes that it would seldom happen, in so many cases of post-mortem examination, instituted any where, without selection, that this organ should be found so thoroughly entire, and at Hong-Kong in like manner, he remarks, "it very seldom happens that any alteration from normal formation can be detected."

We have confined our observations hitherto to fever and dysentery, because they are the chief causes of sickness and mortality among the force in China; indeed, all other diseases are comparatively rare and of little intensity. Notwithstanding the difference between the temperature of the summer and winter months, and the diurnal variations of the thermometer during the latter being considerable, the sailors enjoyed a great exemption from catarrhal and rheumatic affections. Phthisis also has been scarcely met with there; a fact of considerable interest when viewed in connexion with the immunity enjoyed from it by the troops in India. Dr. Wilson seems inclined, although he does not positively assert it, to attribute this to the influence of marsh miasm, remarking that "such exemption is in conformity with what has been noted in other miasmatous districts; it has often been observed that where ague prevails, consumption of the lungs is not rife." This, however, is a question still *sub judice*, and with the fact before us of the great mortality from phthisis among soldiers in the West Indies, where there is no lack either of ague or marshes, we are not prepared to admit the accuracy of this explanation.

A great disposition to the formation of lumbrici was observed; these were voided in great numbers, sometimes even by the mouth, but gave rise to no symptoms by which their existence could be ascertained previous to expulsion.

"It can scarcely be questioned," Dr. Wilson observes, "that the excessive tendency to, and occasional accumulation of them, arises out of the enfeebled, unhealthy condition of the alimentary apparatus—more particularly of the interior membranes; being infested by depraved secretions, and coated with adhesive mucus, it ceases to perform its proper functions adequately, and from the same cause becomes the prolific bed of these creatures....... Their existence is incompatible with a sound state of the living parts in which they are developed; and what is required for their prevention is vigorous healthy action in these parts." (p. 193.)

III. The diseases to which the natives are most subject, besides fevers, cholera, and diseases of the mucous membranes of the lungs and bowels, are stated to be dyspepsia, scrofulous affections, ophthalmia and its results, and diseases of the skin. The causes of these are briefly, but sufficiently, accounted for in the following terms:

"Diet and the habitual practices of the inhabitants contribute powerfully to the frequency of most of their maladies, and are not without influence on them all, in connexion with the malarious influence of the locality. Their food consists almost entirely of vegetables, especially rice, with salted fish badly cured, and often in a semi-putrid state. The large quantities of weak tea drank, and opium-smoking indulged in as often as possible, combine to produce a cachectic condition of the body. Opium, however, is only an occasional indulgence as a luxury; tobacco is considered a necessary, and is universal among both sexes. When otherwise unoccupied, they never drop the tobacco-pipe, smoking from morning till night, and drinking largely, at short intervals, a miserably weak infusion of coarse tea. With innutritious, unwholesome food, and vicious indulgence, cribbed, damp, ill-ventilated apartments, and want of personal cleanliness, will concur to occasion diseases of the lymphatic, cutaneous, and alimentary organs." (p. 22.)

That itch should be almost universal among the natives of all ranks will not appear extraordinary, when we learn that—

"Of water, except in tea-making and some culinary processes, they appear to have no practical knowledge. Bathing, as well as ablution of the person, is unknown; and this is one of the many instances in which they differ from all other people, especially those of the East, inhabiting warm or temperate regions. They go literally unwashed from the cradle to the grave; the only thing in the shape of a substitute observed was a cloth moistened with hot water, and passed lightly over the face and hands by persons of distinction!!"

Truly, baths and wash-houses are needed in China as much as in Whitechapel and St. Giles's.

Among the diseases of the skin under which they occasionally labour, two are deserving of mention. The first is Elephantiasis, or *Barbadoes Leg;* which prevails in China to some extent among the poor, but the cause of it is unknown.

"It consists essentially in inflammation of the subcutaneous cellular tissue which leads to effusion, the effused matter becoming imperfectly organized, occasioning tumefaction, and impairing more or less the motive power of the limb. Repeated attacks of inflammation, effusion, and consolidation produce, if not arrested, in the course of time, generally many years, the highly enlarged, tuberculated, scaly leg, resembling very closely that of an elephant."

The treatment which Dr. Wilson found most beneficial was local abstraction of blood by incision or puncture, according to circumstances.

The other disease to which we alluded is an affection of the same general character with elephantiasis, which attacks the genitals, and is thus described by our author:

"It begins in the scrotum, but soon affects the other parts, producing immense enlargement, agglutination, and confusion of the different tissues, eventually destroying their entire structure. The morbid mass sometimes acquires such dimensions as to descend far below the knees, and requires the limbs to be kept wide apart to make room for its encroachments. In one instance, not at Chusan, the urethra was found by the author eighteen inches below the seat of the tumour. It was discovered only on examination, in front of the tumour's uniform surface, all appearance of the penis being lost. In this affection, as well as that of the limbs, it is probable that early, often repeated, and free incisions, would prove highly beneficial." (p. 25.)

IV. A section is devoted to a short and general sketch of the treatment adopted in the cases of remittent and intermittent fevers, dysentery, and ulcer. We do not intend to follow our author into the details, but would recommend the chapter for the perusal of all—more especially of young medical officers who are likely to be employed in tropical climates. It contains many judicious suggestions, and will make them pause, we trust, before adopting a mere routine system of treatment of these diseases, or concluding that the only hope of success in dysentery is from *throwing in* calomel.

"Routinism," our author well observes, "the besetting sin and bane of medicine, is apt to be indulged in treating tropical diseases, on account of a certain degree of uniformity in their character, and in nothing is the tendency more displayed than in the use of mercury. Calomel, its most favorite, and, on the whole, best preparation, is a remedy which is at once possessed of great power, easy of application by the practitioner, and generally acceptable to the patient. It is, therefore, capable of effecting great good, but is also, and for the same reasons, converted into an instrument of much mischief. Every one has observed its valuable qualities, and occasionally in circumstances which, but for its agency, appeared desperate. Hence has arisen the disposition to ascribe more to it than it has deserved, to carry it further in other cases than was safe, and to employ it in others again, in fact, unlike, though somewhat similar on the surface, to those in which it had proved powerfully remedial. In the use of no medicine is it more necessary than in this to determine what is desired and expected from its operation; and to be fully satisfied, before prescribing it, that the condition that makes such effects desirable, really exists."

The last remark is one deserving of serious consideration by the medical practitioners in this country also, and not in its application to calomel alone, but to every medicine we prescribe. "Cui bono?" is, we fear, a question too seldom asked in writing a prescription.

We have already expressed regret that our author published his work in the form of notes, instead of writing one of a more systematic character. He would then have had an opportunity of lopping off a few redundancies, of bringing his facts more compactly together, of adding materially to the numerical information, and of entering at greater length into several very interesting questions, which he has barely touched upon. The literary execution of the work also would have benefited, as it is natural that notes, written in the bustle of numerous and arduous professional duties, should, in this particular, possess many defects. But in its present shape it is still an excellent and valuable work, and deserving a careful perusal. We have adverted only to a few of the leading topics in it; it contains a large amount of interesting information regarding the topography of Chusan and Hong-Kong; the habits and diseases of the Chinese; the sickness among the troops employed in the expedition, and the causes which produced it; with many useful hints on the means of preserving sailors on shipboard, and of rendering our newly acquired colony in the China seas more salubrious. Into these points we cannot at present enter, but must content ourselves with recommending the work to our readers as one fraught with valuable knowledge, and suggestive of many important reflections. We shall conclude with one more quotation from it. After noticing how little we are able to effect by medicine in many forms of disease, and how often, notwithstanding the boasted improvements of science, we return to the same course of treatment which was adopted by our forefathers, our author asks acutely and answers wisely:—

"To what decision should such things lead? Not to the abandonment or careless pursuit of improvement certainly, but to make us more laborious and rigid than we too often have been, in investigating the fitness of means before they are used, more vigilant in observing, and scrupulous in recording them, and more certain that we do not parade as new and useful, instruments which are old and worthless, or boast of their superior efficacy in cases unlike those in which they have been found unavailing by others. The careful culture of an art, still so indeterminate in many of its applications, cannot be too much encouraged or earnestly pursued; for while so employed, although the immediate object should be missed, it may happen to us, as it happened to the men of old digging for hidden treasure, that we shall turn up something more valuable than that for which we were searching."

---

